# Immune and epithelial responses to textile dyes: the role of chemical structure in toxicity

**DOI:** 10.3389/falgy.2025.1636419

**Published:** 2026-01-09

**Authors:** Lizette M. Cortes

**Affiliations:** Department of Population Health and Pathobiology, College of Veterinary Medicine, North Carolina State University, Raleigh, NC, United States

**Keywords:** disperse Blue 124, Blue 183, Blue 79.1, Blue 1, EpiDerm, epidermal histopathology, epithelial cells

## Abstract

Continuous exposure to textile dyes can result in potential health risks, such as inflammatory and allergic responses. We investigated immunotoxicity, and epithelial responses induced by Disperse Blue 1, 124, 79.1, and 183 when in co-culture with swine peripheral blood mononuclear cells (PBMCs), intestinal porcine epithelial cells (IPEC), and human epidermal skin scaffolds. PBMC cytokine production (IFN-*γ* and TNF-α), cell viability, IPEC gene expression profiles (by Nanostring analysis) and histopathology of human epidermis were evaluated. Disperse Blue 124 strongly increased pro-inflammatory cytokines without significant cytotoxicity, suggesting high sensitization potential. Contrarily, Disperse Blue 1 exhibited high cytotoxicity despite moderate cytokine production. Nanostring analysis revealed prominent epithelial inflammation (CCL20 upregulation) and compromised barrier integrity (CLDN-4) with Blue 1 and Blue 124, but not Blue 79.1 and 183. Histopathology further confirmed epidermal damage, with Blue 1 and 124. Therefore, dye-induced effects correlated with chemical structure, molecular weight, hydrophobicity, and functional groups, supporting the hypothesis that structural properties influence toxicity and absorption.

## 1 Introduction

Synthetic textile dyes are organic compounds widely used to confer color to fabrics and garments. Disperse dyes are particularly important for dyeing synthetic fibers like polyester and acetate (1). While essential to the textile industry, many dyes have been implicated as environmental and human health hazards (2–5). Disperse dyes are characterized by low water solubility and high affinity for hydrophobic fibers, properties that also facilitate skin penetration (1). Synthetic disperse dyes used in textiles have potential allergenic and inflammatory risks. In fact, disperse blue dyes were selected as contact allergens for the year 2000 (4). The clinical relevance of allergic contact dermatitis from disperse blue dyes is estimated to be up to 70% (3). Structural factors such as molecular size, hydrophobicity, and functional groups may influence dye toxicity and skin absorption (1). Azo and anthraquinone disperse dyes are of special concern due to their structural features, which include reactive functional groups capable of interacting with biological macromolecules (1). Despite their widespread use, comprehensive toxicological data on individual disperse dyes remain limited. This study aimed to investigate the viability and immune responses of swine PBMCs, IPEC cells and human skin scaffold responses to four class category representative disperse dyes with blue hues: Disperse Blue 1, Blue 124, Blue 79.2, and Blue 183, testing the hypothesis linking chemical structures and biological activity. Chemical structure, molecular weight, and functional groups of each dye were compared against biological outcomes.

## 2 Materials and methods

Swine PBMC and IPEC cultures were exposed to environmental relevant exposure levels (according to Cortes et al. 2025) of four disperse dyes (Blue 1, Blue 124, Blue 79.1, Blue 183). Cytokine profiling, cell viability, gene expression analysis, and human epidermal skin scaffold histology were assessed to evaluate immunotoxicity and epithelial effects.

### 2.1 Ethics statement

The human skin scaffold model used in this study was commercially obtained from a certified supplier (MatTek Corporation, EpiDerm). All tissues were derived from de-identified human donors, provided under compliance with ethical standards and the Declaration of Helsinki. No living human participants were recruited, and no identifiable information was used. As such, institutional review board (IRB) approval and informed consent were not required. Swine PBMC isolation was done in accordance with and approved by the North Carolina State University Institutional Animal Care and Use Committee (IACUC ID# 18-084-B).

### 2.2 Dyes in cell cultures

The following disperse (D) dyes were used in this study: D. Blue 1 (Sigma 215643-5G), D. Blue 124 (Sigma 21620-5G), D. Blue 79.1 (Sigma 75497-74-4), and D. Blue 183 (ACM2309946). Solubility was tested in different solvents and at different concentrations to determine the optimal solubility of all dyes and with the highest cell viability and low toxicity from the solvent. Acetone and DMSO (Corning 25-950-CQC) were selected as solvents. The dissolved dyes were centrifuged to remove insoluble particles or possible contamination for the cell culture.

### 2.3 Cell viability and analysis of the T-cell response

Swine naive PBMC viability was measured via flow cytometry, and they were also stained with antibodies for cytokines TNF-α (Biolegend 502936) and IFN-γ (BD Biosciences 559812). Precisely, PBMCs from adult pigs, isolated by density centrifugation using Ficoll-Paque-Premium (GE Healthcare, Uppsala, Sweden) in SepMate tubes (StemCell Technologies, Cambridge, MA, USA) were cultured in 96-well plates at 2 × 10^5^ cells/well in RPMI-1640 (Corning, Corning, NY, USA) supplemented with 10% FBS (VWR, Radnor, PA, USA) and 1× antibiotic-antimycotic (Corning, USA) in the absence or presence of 483.8 ug/well (6) of each textile dye (concentration calculated according to Cortes et al. 2025) and 2.5 μg/mL Con A as a stimulant. After 24 h of culture in the incubator at 37^๐^ C and 5% CO_2_, octuplicate wells from each dye were pooled and stained for flow cytometry analysis using eBioscience Fix/Perm kit (ThermoFisher Scientific, USA) according to the manufacturer's protocol. Antibodies for CD3, clone PPT3 (Southern Biothec), CD4 clone 74-12-4 (BEI Resources) were titrated and used 10 uL of prediluted antibody/well at concentrations ranging from 0.1 µL/well (=20 ng/well) to 0.2 uL/well. PBMC viability was measured by flow cytometry using LIVE/DEAD™ Fixable Near-IR Dead Cell Stain Kit (Invitrogen, USA), according to the manufacturer's protocol. Once PBMCs were gated for single live cells, they were gated on CD3+CD4+ cells to determine expression of TNF-a and IFN- γ. Data was acquired on a Beckman Coulter CytoFlex using the CytExpert software (Beckman Coulter, Brea, CA, USA). Data analysis was performed using FlowJo v10.5.3 (FLOWJO LLC, Ashland, OR, USA).

### 2.4 Gene expression profiling using nanostring technology

To answer the question if the different textile dyes lead to an overall downregulation or upregulation of proteins involved in epithelial inflammation or compromised barrier integrity, IPEC cells exposed to dyes at a concentration of 2,424 µg/mL in 15 mL of culture medium, for a total of 36.36 mg per T75 flask (6) and exposed to the different dyes in DMEM-F12 supplemented with 10% FBS (VWR, Radnor, PA, USA) for 24 h (this time was sufficient to observe meaningful transcriptional changes). Dye uptake was confirmed by microscopy. After detaching the cells and a round of washes, RNA from IPEC cells was isolated using (Zymo RNA purification kit, USA) according to the manufacturer's protocol. RNA preps were diluted to a concentration of 20 ng/μL. Later, IPEC cells underwent molecular profiling using the nCounter Analysis system. Using a uniquely designed codeset (nCounter XT CodeSet Gene Expression Assay NanoString Technologies Seattle, WA), hybridization buffer was added to the reporter CodeSet to create a master mix. An aliquot of the master mix was mixed with 5 μL of each RNA sample, followed by the capture probe. The samples were placed at 65 °C in a pre-heated thermal cycler for at least 18 h. One sample per lane was loaded and then processed in the nCounter (NanoString Technologies) according to instructions and using 555FOVs counts. CLDN-4, VEGF, CCL-20, CXC9, IL-1RA, IL-16 were analyzed with the custom nanostring panel for each dye.

### 2.5 Epiderm cultures

Human full thickness epidermal skin scaffolds (Mattek, USA) derived from differentiated 3D tissue model from normal human epidermis (EFT-412-BW) under air-liquid interface (ALI) were first upon received, equilibrated with hydrocortisone free media with 2.5 mL in 6 well plates overnight (EFT-400-MM-HCF). Considering that a T-shirt is dyed with ∼2 g of dye for a surface of∼4,948 cm^2^∼and that the surface of the Epiderm tissues is 1 cm^2^, we used 404 ug of the mentioned dyes (6) and incubated them with the cultures for 24 h of each textile dye (concentration and time calculated according to Cortes et al. 2025). Dye uptake was confirmed by microscopy. Tissues were then harvested, and histological sections were stained for H&E and were scored for epidermal damage (scale 0–3) by a blinded licensed pathologist. The scoring was done as follows: Epidermis scores 0 = Normal thickness with differentiated layers, 1 = Normal thickness with differentiated layers and scattered epithelial apoptosis, 2 = Loss of one or more layers of epidermis with scattered epithelial degeneration or apoptosis, 3 = Only stratum basal and stratum corneum with scattered epithelial degeneration or apoptosis.

### 2.6 Statistical analysis

Data are expressed as mean ± standard deviation (SD) from at least three independent experiments. For comparisons involving more than two groups, a one-way analysis of variance (ANOVA) was performed. Where ANOVA indicated significant differences, Dunnett's multiple comparisons test was used to compare each textile dye treatment group to the media control. A *p*-value of <0.01 was considered significant. All analyses were done with GraphPad Prism version 10.2.3 (San Diego, CA, USA).

## 3 Results

### 3.1 *In vitro* assays and cytokine production

**Figure 1:**
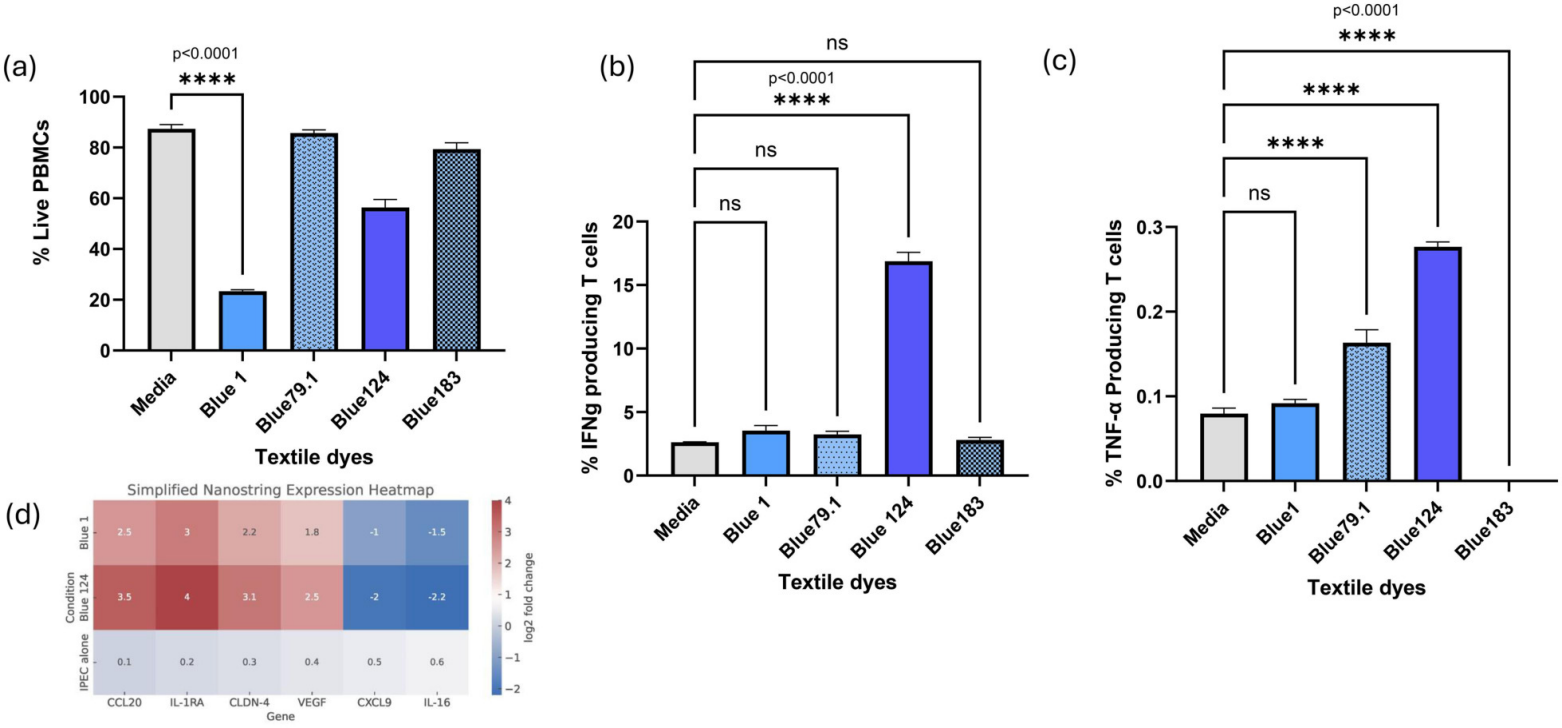
**(a)** PBMC viability, percentage of live PBMCs and **(b,c)** cytokine production after dye exposure. Bar graphs showing percentage of TNF-α and IFN-γ producing T cells after 24h dye exposure. Blue 124 induced the highest cytokine production; Blue 1 caused marked significant reduction in cell viability. **(d)** Nanostring gene expression heatmap of IPEC cells after dye exposure. Heatmap depicting differential expression of key immune and stress response genes. Strong upregulation of CCL20, IL-1RA, CLDN-4, and VEGF was observed for Blue 1 and Blue 124. Created with BioRender.com.

PBMC assays revealed distinct cytokine and viability profiles: Disperse Blue 124 (MW: 377.42 g/mol, high hydrophobicity, complex aromatic functional groups) strongly activated IFN*γ* and TNF-α production, maintaining moderate viability. Blue 1 (MW: 268.27 g/mol, anthraquinone structure, moderate hydrophobicity) displayed lower cytokine production but high cytotoxicity. Blue 79.1 (MW: 625.4 g/mol, intermediate hydrophobicity and structure complexity) and Blue 183 (MW: 473.32 g/mol, stable azo dye, lower hydrophobicity) elicited none to low immune responses with minimal cytotoxicity (Tables 1, 2; Figures 1a–c).

**Table 1:** Chemical properties of the textile dyes used.

Textile dyes	Chemical formula	Molecular weight (g/mol)	Functional groups	Chemical structure
Disperse Blue 1	C_14_ H_12_ N_4_ O_2_	268.27	Amino, Anthraquinone	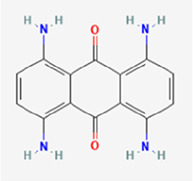
Disperse Blue 124	C_16_H_19_N_5_O_4_S	377.42	Azo, Nitro, Thiazole, Amine	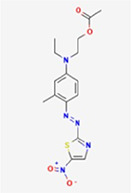
Disperse Blue 79.1	C_23_ H_25_BrN_6_O_10_	625.4	Azo, Nitro, Bromo, Nitro, Cyano, Amine	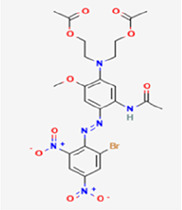
Disperse Blue 183	C_20_H_21_ BrN_6_O_3_	473.32	Azo, Bromo, Nitro, Cyano, Amine	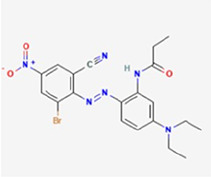

Lists of molecular weight, chemical formula, functional groups, and structure type for each dye. Created with BioRender.com.

**Table 2:** Summary of biological responses across test systems.

Textile dyes	PBMC IFN-*γ*/TNFα	IPEC response (Nanostring)	Epidermal damage score	Overall toxicity profile
Disperse Blue 1	Moderate/Low	High (CCL20, CLDN-4 ↑)	2	Moderate Toxicity
Disperse Blue 124	High/High	Very high (Broad inflammation)	3	Potent Sensitizer/Irritant
Disperse Blue 79.1	Low/Low	Mild	1	Low toxicity
Disperse Blue 183	Low/Low	Mild	1	Low toxicity

Comparative results of PBMC cytokine responses, IPEC-Nanostring gene expression, and epidermal scaffold damage scores. Blue 124 showed the highest inflammatory and damaging profile. Created with BioRender.com.

### 3.2 Nanostring analysis

Nanostring analysis for IPEC cells highlighted significant CCL20, IL-1RA, CLDN-4, and VEGF upregulation for dyes Blue 1 and Blue 124, correlating with their chemical features favoring cellular stress and inflammation. CLDN-4 and VEGF were upregulated, and this could suggest alterations in epithelial barrier integrity and angiogenesis-related processes (Table 2; Figure 1d). CXCL9 and IL-16 were notably downregulated, across treatments with dyes Blue 1 and Blue 124, which could have implications for immune signaling and inflammation, especially in barrier tissues like the gut or skin indicating potential suppression or regulation of these cytokines suggesting immunomodulatory effects linked to structural characteristics. CXCL9 is a chemokine induced by IFN- γ that attracts activated T cells. Its downregulation could imply reduced T cell recruitment to the epithelial barrier and potential dampening of the Th1 mediated inflammation. IL-16 is a chemoattractant for CD4+ T cells involved in modulating epithelial cell signaling. Its downregulation could imply reduced CD4+ T cell chemotaxis and local immune activation, as well as possible reduced immune surveillance at the epithelial cell surface. It could also indicate epithelial stress or damage.

### 3.3 Gross pathological changes

In all slides, the dermis lacked well-formed collagen bundles; however, scattered spindle shaped cells (likely fibroblasts) were present. Several sections had acutely affected epithelium at the margins of sections. This was suspected to be from tissue handling. Histopathological analysis of human skin scaffolds revealed consistent trends (Table 3). Blue 124 induced the highest epidermal damage (Score = 3), with extensive keratinocyte necrosis and structural disorganization. Blue 1 produced moderate damage (Score = 2), while Blue 79.1 and Blue 183 resulted in only mild focal necrosis (Score = 1). These results strongly correlated with cytokine induction and gene expression data. Histology confirms that both immune activation and cytotoxicity in PBMC/IPEC models translate into visible tissue damage in skin. The correlation between dye structure and epidermal damage may strengthen the hypothesis that chemical properties (size, hydrophobicity, reactivity) are central to skin toxicity. The damage pattern (e.g., vacuolization, nuclear fragmentation) matches what we expect from chemical irritants or sensitizers.

**Table 3:** Histopathology of human epidermal skin scaffolds.

Textile dyes	Epidermal damage score	Histological description	H & E
Disperse Blue 1	2	Vacuolated keratinocytes, pyknotic nuclei, cytoplasmic eosinophilia	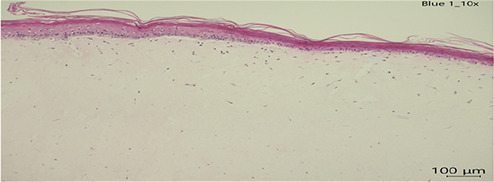
Disperse Blue 124	3	Lifted/Partially intact epidermis, nuclear fragmentation, widespread cytoplasmic eosinophilia	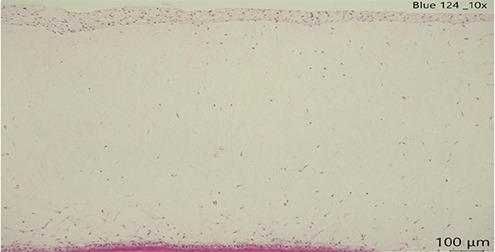
Disperse Blue 79.1	1	Well-differentiated keratinocytes, marginal necrosis, nuclear fragmentation	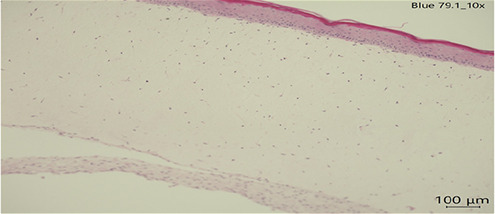
Control	1	Well-differentiated keratinocytes, marginal necrosis, nuclear fragmentation	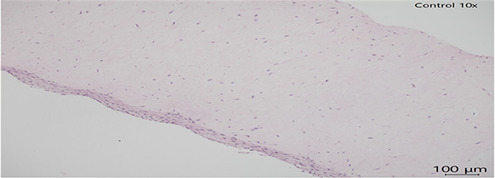

Representative histology images (H&E staining) showing dye-induced epithelial damage. Blue 124 caused severe keratinocyte necrosis; Blue 1 caused moderate damage; Blue 79.1 and Blue 183 (data not shown) showed minimal effects. Created with BioRender.com.

### 3.4 Statistical analysis

A one-way ANOVA revealed a significant effect of textile dye treatment on IFN-*γ* and TNF-α production (*p* < 0.0001). *Post-hoc* Dunnett's test showed that Blue 124 significantly increased IFN-*γ* and TNF-α+ T cells compared to media control (*p* < 0.0001) and a significant reduction of cell viability for D. Blue 1 (*p* < 0.0001).

## 4 Discussion

Our findings suggested dye- immune activation, epithelial stress, and epidermal injury linked to chemical structures. Low molecular weight, structural complexity, and hydrophobicity correlated with enhanced inflammatory response and potential sensitization (Blue 124), whereas simpler structures (Blue 1) correlated with cytotoxic effects, possibly due to facilitated cellular entry and reactive intermediate formation. Disperse blue 1 is an older dye, less stable and more toxic than disperse blue 183 (1). Disperse Blue 183 has a significantly higher molecular weight, reflecting its more complex structure. Disperse Blue 1 has been historically used in textiles and cosmetics but is now restricted due to toxicity concerns. Disperse Blue 183 is favored in modern applications for its stability and safety profile (1).

Disperse Blue 124 displayed the highest TNF-α and IFN-*γ* induced production. TNF-α tends to have an inhibitory effect on proliferation of PBMCs in pigs, it can induce cell arrest, apoptosis in activated T or B cells, and suppression of mitogen- induced proliferation like with Con A (7). The inhibitory effect could be via its receptor TNF-αR1, activating pro-apoptotic pathways or modulating NF-KB to skew cells to get activated however without proliferation (7). When PBMCs come in contact with azo or disperse dyes, it has been reported that they can get activated as long as the dye is a hapten that becomes antigenic by binding to proteins (8). The production of IFN-*γ* suggests that we have a type-Th1 immune response usually associated with inflammation and that the dye could be immunostimulatory and possibly immunotoxic (8). IFN-*γ* drives the production of pro-inflammatory cytokines like TNF-α and reactive oxygen species. It can have a bystander effect or immune mediated cytotoxicity. Repeated exposure to IFN-*γ* inducing dyes could contribute to skin or respiratory hypersensitivities that could lead to contact dermatitis (8).

Nanostring analysis highlighted significant CCL20, IL-1RA, CLDN-4, and VEGF upregulation for dyes Blue 1 and Blue 124 and downregulation for CXCL9 and IL-16. CCL20 upregulation is associated with epithelial inflammation and immune cell recruitment, often activated via NF-κB or MAPK signaling pathways (9). Future studies will aim to characterize the effect of these textile dyes on this pathway. CXCL9 downregulation could indicate suppression of certain Th1-immune responses or modulation in chemotactic signaling (10). IL-16 is involved in T cell migration and inflammation (11), its downregulation may suggest immune modulation by textile dyes. CLDN-4 changes could indicate altered epithelial barrier function, possibly due to toxicity or cell stress (12). Blue 124 showed high IFN-γ and IFN-*γ* in PBMC, correlating well with strong epithelial inflammation indicated by CCL20 upregulation. Blue 1 showed lower cytokines but high cytotoxicity (low proliferation), consistent with epithelial damage/inflammation (CCL20 high, CXCL9/IL-16 downregulated). The downregulation of CXCL9/IL-16 could indicate that dyes like Blue 1 and Blue 124 could suppress local immune signaling in epithelial cells. This could reflect toxic suppression of immune genes due to cell stress or mitochondrial dysfunction as seen in the previous paper by Cortes and Vinueza 2025. This could also be an attempt by epithelial cells to limit inflammation in response to chemical irritation. This gives us a dye-specific effect that selectively reduces Th1 attracting chemokines and shifts the immune response. This similar effect has been observed in models of epithelial injury caused by infection or chemical exposure (13). Disperse textile dyes like blue 124 have been shown to cause positive reactions in skin patch tests (2, 3).

Although the blue dyes tested in this study are functionally similar, they are chemically distinct in origin and immunological behavior (1). They seem to differ in toxicity, metabolism and sensitization potential due to their structural differences (1). Results from PBMCs, IPECs, and human epidermal scaffolds were in strong agreement, reinforcing the predictive value of *in vitro* and *ex vivo* models. However, although this 3D scaffold model was useful, it does not fully recapitulate *in vivo* skin with intact vasculature and immune cell populations. The dye concentrations used in this study were derived from previous work; however, an important consideration is that real-world exposure involves percutaneous dosing during garment wear, influenced by dye leaching kinetics from the textile matrix into sweat and skin. These percutaneous doses depend on the duration of skin-fabric contact, skin condition, body location, and individual skin barrier integrity among others. The leaching kinetics could depend on the dye-fiber binding strength, chemical structure and hydrophobicity as well as local environmental conditions such as humidity and temperature (14, 15).

## 5 Conclusion

Chemical structure, molecular size, hydrophobicity, and functional groups may seem to influence textile dye toxicity and skin absorption. Additional studies are needed to clarify these structure-activity relationships. While our findings raise the hypothesis that the chemistry of certain dyes could play a role to trigger immune responses associated with atopic diseases, such implications require further investigation. Comprehensive toxicological assessments that incorporate structure-activity relationships are needed to guide the textile dye industry in selecting dyes that are less likely to trigger immune responses.

## Data Availability

The raw data supporting the conclusions of this article will be made available by the authors, without undue reservation.
